# Expression Profiling of Autism Candidate Genes during Human Brain Development Implicates Central Immune Signaling Pathways

**DOI:** 10.1371/journal.pone.0024691

**Published:** 2011-09-15

**Authors:** Mark N. Ziats, Owen M. Rennert

**Affiliations:** 1 Laboratory of Clinical and Developmental Genomics, National Institute of Child Health and Human Development, National Institutes of Health, Bethesda, Maryland, United States of America; 2 Medical Scientist Training Program, Baylor College of Medicine, Houston, Texas, United States of America; 3 Department of Physiology, Development and Neuroscience, National Institutes of Health-University of Cambridge Biomedical Scholars Program, Cambridge, United Kingdom; University of Georgia, United States of America

## Abstract

The Autism Spectrum Disorders (ASD) represent a clinically heterogeneous set of conditions with strong hereditary components. Despite substantial efforts to uncover the genetic basis of ASD, the genomic etiology appears complex and a clear understanding of the molecular mechanisms underlying Autism remains elusive. We hypothesized that focusing gene interaction networks on ASD-implicated genes that are highly expressed in the developing brain may reveal core mechanisms that are otherwise obscured by the genomic heterogeneity of the disorder. Here we report an *in silico* study of the gene expression profile from ASD-implicated genes in the unaffected developing human brain. By implementing a biologically relevant approach, we identified a subset of highly expressed ASD-candidate genes from which interactome networks were derived. Strikingly, immune signaling through *NFκB*, *Tnf*, and *Jnk* was central to ASD networks at multiple levels of our analysis, and cell-type specific expression suggested glia—in addition to neurons—deserve consideration. This work provides integrated genomic evidence that ASD-implicated genes may converge on central cytokine signaling pathways.

## Introduction

The Autism Spectrum Disorder is a heterogeneous neurodevelopmental syndrome defined by impairments in communication, social interaction, and restricted or stereotyped patterns of behavior. ASD is the most heritable of the common neuropsychiatric conditions with estimates approaching 90% in monozygotic twins, 10% in dizygotic twins, and recurrence risk in siblings 10–100 times the general population [1], [2], [3], [4]. Moreover, approximately 10–20% of ASD cases are associated with recognizable syndromes of known etiology—representing a large number of rare alleles [5]. With recent advances in comparative genomic hybridization (CGH), approximately 40% of patients with a diagnosis of ASD will have a detectable genomic aberration [6]. However, this genetic etiology is complex and likely involves gene-gene, gene-environment, and epigenetic interactions, reflecting the overlying broad clinical presentation of ASD. This is evidenced by the less than 100% penetrance in identical twins, the discordance in heritability between mono- and dizygotic twins, and the considerable variability within pedigrees [7], [8]. Furthermore, the clinical phenotype and underlying genetics of the syndromic forms of ASD are extremely varied, and differences in manifestations of the three core symptoms are observed even within a specific diagnostic entity. Moreover, ASD shares considerable clinical and genetic overlap with other neuropsychiatric disorders such as schizophrenia and mental retardation [9], and ASD patients have significantly increased neurologic co-morbidities like hypotonia, tics, and epilepsy [10]. In fact, many of the same gene mutations have been found to predispose to more than one of these neurodevelopmental disorders [11], [12].

Consequently, the approximately 60% of non-syndromic ASD cases without an identifiable structural variation (here defined as “intrinsic” Autism) represent a broad clinical spectrum with strong genetic underpinnings that have proven exceedingly difficult to define. Much work has attempted to elucidate the molecular genetics underlying intrinsic Autism, with many linkage, functional, and genome-wide association studies (GWAS) having implicated more than 200 loci to date [13], [14], [15]. Additionally, copy number variation (CNV) and cytogenetic analysis have further identified many chromosomal hot spots in ASD [16], [17]. It is apparent from these studies that many different loci, each with a presumably unique yet subtle contribution to neurodevelopment, underlie the phenotype of ASD. These observations have prompted a shift in the paradigm of ASD genetics away from a common disease/common variant model, to one that recognizes the contribution of rare variants [5], [9]. Because of this great clinical and genetic heterogeneity, attempts to identify a common molecular pathology for ASD have remained elusive, and as a result, diagnosis and treatment are non-specific and suboptimal.

Although Autism currently lacks any unifying principles at the genetic and molecular levels, both human and animal studies have begun to demonstrate that disruption of synaptogenesis and improper connectivity of local and distant brain networks likely underlie the cellular pathophysiology responsible for the broad ASD phenotype [18], [19]. Multiple different brain regions have been implicated in both post-mortem and neuroimaging studies, notably the prefrontal and temporal cortices, and the cerebellum [20]. Histological analysis has revealed increased cell densities, changes in synaptic spine morphology, mini-columnar disorganization, and glial activation [21]. Despite these observations, the mechanism(s) responsible for this “disconnection” phenotype remains obscure, as a complex interplay between diverse cell types and functions modulate the developing network architecture in both a temporal and spatially regulated manner [22], [23], [24]. A main question in ASD research has become, then, how to reconcile the genetic and phenotypic heterogeneity with the apparent synaptic network abnormalities underlying the broad ASD phenotype.

A proposed unifying explanation for this dichotomy posits that differences in gene expression in the developing brain could explain how many genes, each with a different contribution to proper formation of brain circuitry, could result in a single disorder with neural network dysfunction at its core [25], [26]. This model is underscored by the prototypical Autism Spectrum Disorder, Rett Syndrome, in which mutations in the *Mecp2* gene result in global dysregulation of the transcriptome [27]. Moreover, it has been shown that mutations in *Mecp2*—a transcriptional repressor—result in aberrant expression at many ASD-implicated loci [28]. To investigate this model, however, requires gene expression profiling of ASD-candidate genes in developing human brain tissue. To date, a number of studies have investigated gene expression in ASD (for review see [29]), with three examining ASD brain tissue on a genome-wide scale [30], [31], [32]. However, no study explicitly describes the transcriptional profile of ASD-implicated genes, and by necessity, all were limited in developmental time points and brain regions investigated.

To investigate more thoroughly the notion that differences in expression of ASD-implicated genes underlies the complex genomics of the disorder, we hypothesized that focusing gene interaction networks on ASD-implicated genes with high expression in the developing brain may reveal core mechanisms that are otherwise obscured by the heterogeneity of all implicated loci. To do this, we mined the NIMH Transcriptional Atlas of Human Brain Development [33] for all genes implicated in ASD that are included in the database AutDB [34]. The NIMH Atlas contains next-generation RNA sequencing data from 16 normal human brain regions, and spans 21 weeks gestation through 40 years of age. We devised a biologically-driven computational approach to analyze differential expression across regions and development, and assessed cell-type specific expression using the Human Protein Atlas [35]. We discovered distinct molecular interaction networks using an enriched set of highly expressed genes, which implicated canonical immune signaling pathways at multiple levels of analysis as central to ASD.

## Results

### Evaluating Differential Expression in NIMH Transcriptional Atlas of Human Brain Development

The NIMH Atlas reports the normalized reads per kilobase of exon model per million mapped reads (RPKM) units [36]; whereas primary RNA-seq analysis pipelines have the advantage of using raw read counts for statistical evaluation of differential gene expression. Thus, we first established a qualitative differential expression methodology that could directly interpret RPKM values with consistency and validity across different brain regions and time points. This allowed us to identify a subset of genes that were highly expressed directly from RPKM data.

To achieve this, we examined the expression profile of the top 15 genes determined by Hsiao *et al* as constantly expressed from 59 different whole-genome microarrays in 19 different tissue types [37]. For 11 of the 15 genes there was consistency in expression across developmental time points and in different brain regions (Fig. S1). To validate our approach further, we selected at random 10 canonical housekeeping genes representing 10 different cellular processes [38]. We observed consistent expression for all 10 of these genes across brain regions and time points (Fig. S2). This resulted in a total of 21 housekeeping genes with constant expression (11 from Hsiao *et al* and 10 canonical), which we used to define normal biological variance in the NIMH Atlas. To stratify the NIMH Atlas data, we grouped expression values into quintiles (<20 RPKM, 20–40 RPKM, 40–60 RPKM, 60–80 RPKM and >100 RPKM), as previously reported for microarray expression data in brain [39]. Of the 21 constantly expressed housekeeping genes, we noted they all vary within three consecutive quintile tiers. Based on these results, we concluded that genes crossing more than three tiers were significantly differentially expressed, as opposed to exhibiting normal biological variation. This initial approach demonstrated that reported RPKM values could be used qualitatively to assess differences in gene expression levels.

Notably, expression values at the 6 mo time point were considerably lower for almost all genes and brain regions. This may be a function of lowered CNS transcriptional activity at this age, however a systematic error in sequencing is also likely. Since we were interested in highly expressed genes, we were not concerned this would introduce false-positive results into our subsequent analysis.

### Expression of Brain-specific markers

We analyzed genes of brain-specific markers (adhesion/elastic/filament proteins) with intermediate expression to further validate our method and gain insight into cell-type specific expression across brain regions and during different developmental time points. As seen in Figure S3, Keratin and Desmin—markers of epithelia and muscle, respectively—were not expressed as expected. Neurofilament (*Nefl*), a neuron-specific maker, showed high expression in most brain regions after 24 weeks gestation (wg). Notably, expression of *Nefl* was significantly lower in the cerebellum, which is consistent with our observation at the protein level (see below). Expression of Glial Fibrillary Acid Protein (*Gfap*), an astrocyte-specific intermediate filament, also showed high expression in all brain regions beginning at the fourth postnatal month, although markedly later in development than *Nefl*. Interestingly, Vimentin, a marker of mesenchyme-derived cells, exhibits a differential expression pattern with very high expression in the early developing brain (24 wg–4 mo). This may be a reflection of invading microglia, which are of mesenchymal origin and known to enter the developing brain during early embryogenesis [40], and/or it may relate to the laying down of the vasculature and extracellular matrix early in development.

### Expression Profile of genes implicated in ASD, Epilepsy, and Schizophrenia

**Figure 1:**
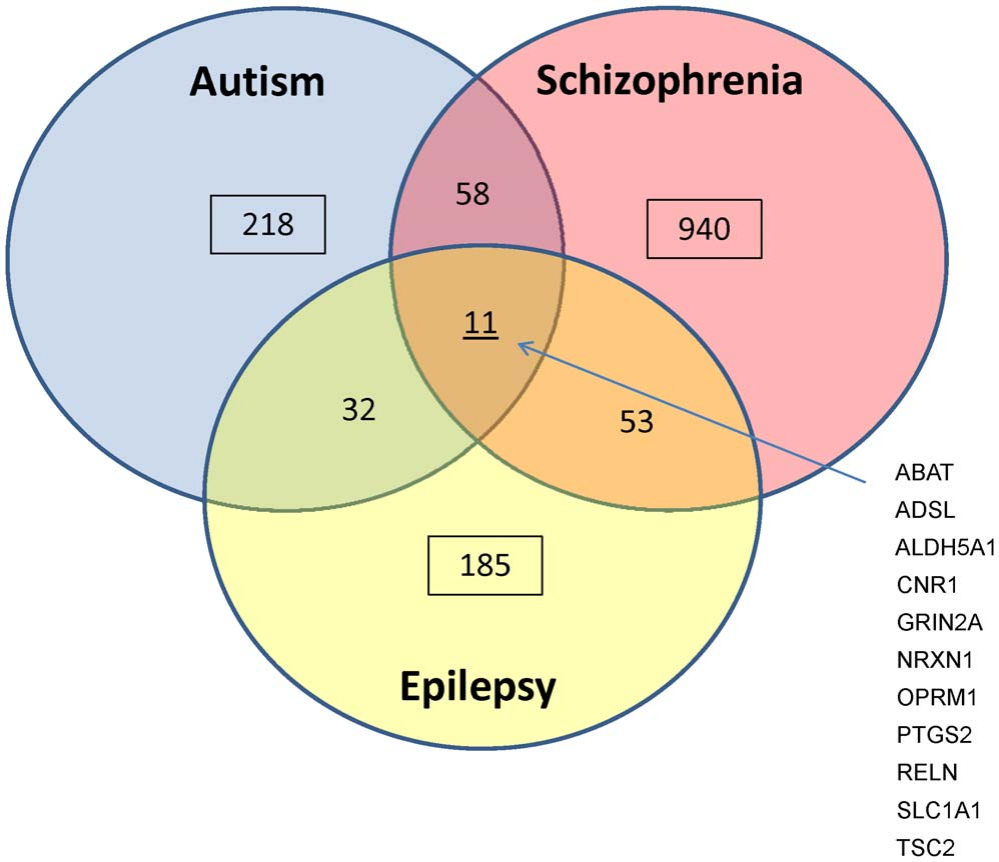
Summary of all genes analyzed from AutDB, CarpeDB and SZGene. Number of genes and genes shared between disorders indicated.

We parsed the NIMH database for all genes implicated in Autism that are described in the database AutDB (Table S1 and Methods). To strengthen our approach and investigate the overlapping genetic and clinical aspects of Schizophrenia and Epilepsy with ASD, we also investigated all genes implicated in these disorders, which are cataloged in the databases SZGene and CarpeDB, respectively (Table S1). Non-redundant, protein-coding loci that were present in the NIMH atlas were included in this study, as summarized in Figure 1. Only 11 genes are shared by all three disorders. Gene ontology (GO) enrichment analysis of these 11 overlapping genes as opposed to all genes implicated in all three disease databases yielded many significant pathways mainly involved in the response to external stimuli and GABA metabolism (Table 1).

**Table 1:** GO enrichment analysis of the 11 genes shared by Autism, Schizophrenia, and Epilepsy.

GO Term	Description	P-value
GO:0042220	response to cocaine	1.11E-05
GO:0014073	response to tropane	1.11E-05
GO:0014070	response to organic cyclic compound	6.99E-05
GO:0009450	gamma-aminobutyric acid catabolic process	7.68E-05
GO:0051259	protein oligomerization	1.22E-04
GO:0043279	response to alkaloid	1.75E-04
GO:0009605	response to external stimulus	1.85E-04
GO:0051260	protein homooligomerization	1.99E-04
GO:0009448	gamma-aminobutyric acid metabolic process	2.29E-04
GO:0032103	positive regulation of response to external stimulus	6.05E-04
GO:0010042	response to manganese ion	7.57E-04
GO:0042135	neurotransmitter catabolic process	7.57E-04
GO:0031622	positive regulation of fever generation	7.57E-04
GO:0031620	regulation of fever generation	7.57E-04
GO:0031650	regulation of heat generation	7.57E-04
GO:0031652	positive regulation of heat generation	7.57E-04
GO:0009607	response to biotic stimulus	8.35E-04

We constructed expression heatmaps for all genes by brain region and time-point by assigning each RPKM expression value to one of five quintiles, and then grouping genes into five expression tiers (see Methods; Tables S2, S3, S4). Strikingly, for each of the three disease sets more than 55% of genes were never expressed above 20 RPKM, with the majority of these less than 5 RPKM (Table 2). For ASD candidate genes, greater than 70% were not expressed highly in each brain region. In each region, a large percentage of ASD-implicated genes had no detectable transcription (<1 RPKM). For instance, in the hippocampus 46 out of 219 (21%) ASD-implicated genes had no detectable transcripts. Similar proportions were not detected in the cerebellum (52/219 or 24%) or dorsolateral prefrontal cortex (40/219 or 18%). While it is possible that these loci still have functional roles in ASD genomics via *cis*-regulation or other mechanisms, we reasoned that their inclusion in protein-interaction networks might obscure more prominent molecular mechanisms underlying ASD.

**Table 2:** Summary of differential gene expression across all brain regions.

Region	% of genes less than 20 RPKM			% of genes in Top 3 Tiers		
	Autism	Epilepsy	Schizophrenia	Autism	Epilepsy	Schizophrenia
DLPC	71%	59%	67%	7%	18%	16%
VLPC	73%	59%	67%	7%	17%	16%
MPC	77%	59%	69%	7%	18%	15%
OFC	74%	58%	69%	6%	20%	16%
Motor	74%	61%	68%	6%	18%	16%
PS Temp	74%	57%	69%	6%	19%	16%
IL Temp	73%	59%	68%	8%	21%	15%
Hippo	79%	63%	70%	5%	15%	14%
Amygdala	74%	56%	66%	5%	19%	16%
Striatum	73%	58%	67%	6%	19%	16%
Cerebellum	77%	62%	67%	8%	14%	15%

It is of note that the cerebellum and frontal cortex contained the greatest number of highly expressed “Autism genes” and the temporal cortex had the greatest number of “Epilepsy genes,” whereas Schizophrenia gene expression distributed more evenly throughout the brain. While much work in Autism has focused on the hippocampus as a potential epicenter of pathology, we found the developing hippocampus had the fewest ASD candidate genes expressed at high levels, and none were specific for the hippocampus. Conversely, the cerebellum contained a unique set of six Autism candidate genes that were not highly expressed in any other brain region. These included the canonical neurodevelopmental genes *Nlgn3* and *Reln*, two cell adhesion molecules, and 7-dehydrocholesterol reductase. This is intriguing since multiple imaging studies have implicated the cerebellum in the pathogenesis of Autism [41]. The NIMH Atlas parcels the frontal cortex into four subregions, yet the expression profile of ASD genes between them was similar. Only one gene (*Gabrb3*) was specific to the frontal cortex, and it was only present at high levels in the ventrolateral prefrontal cortex. Interestingly, this gene lies in the 15q11–13 imprinted region implicated in Prader-Willi and Anglemen Syndromes, and is one of the most reproducible loci identified in ASD genome-wide association studies [42].

**Figure 2:**
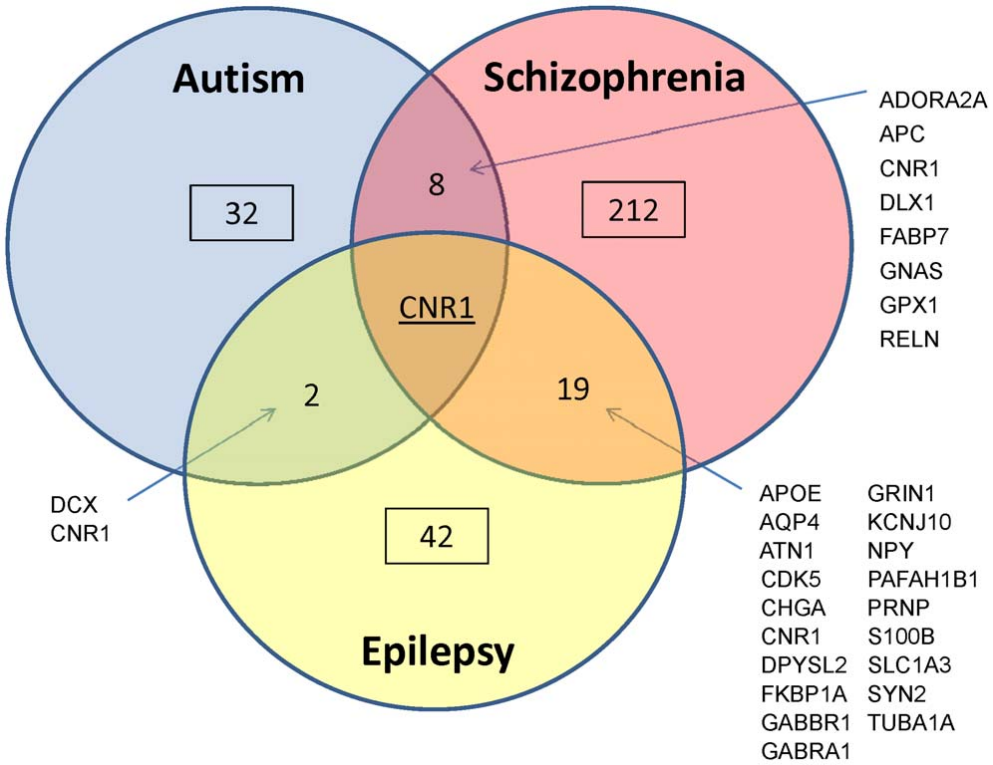
Summary of the subset of highly expressed genes identified from our analysis.

For the remainder of our analysis, we focused on genes in the top three expression tiers (at least one time-point >60 RPKM) as genes that are significantly highly expressed as compared to all ASD-implicated genes (based on our “housekeeping gene” results). This yielded 32 genes for Autism, 42 for Epilepsy and 212 for Schizophrenia (Fig. 2). Autism shared eight highly expressed genes with Schizophrenia, and only two with Epilepsy (*Dcx* and *Cnr1*). GO enrichment of these nine shared genes did not identify any significant pathways. There was only one gene—Cannabinoid Receptor 1 (*Cnr1*)—implicated in all three disorders that is highly expressed in the developing brain. *Cnr1* expression is high mainly during gestation, and is most pronounced in the cerebellum and amygdala (Fig. S4).

Nine Autism genes were highly expressed in all brain regions examined. These nine genes (*Fabp7, Gnas, Gpx1, Hnrnph2, Hras, Pdzd4, Rpl10, Sez6l2*, and *Tspan7*) had considerably higher expression than all other ASD genes (over 500 RPKM in many instances, Fig. S5). Their temporal expression profiles were mostly constant across developmental stages, except for *Fabp7*, which exhibited drastic differential expression. *Fabp7* was expressed much higher than the other eight genes during almost all time-points, but was highest during the two gestational time points. Interestingly, *Fabp7* (Fatty acid binding protein 7) is known to interact with *Notch* in radial glia during development [43], and we subsequently found it to only be expressed in glia (see below). The temporal expression of the other 32 highly expressed genes varied considerably, but was biased toward high expression in the early time points analyzed (Table S5).

Gene ontology enrichment of the 32 highly expressed Autism genes revealed four new GO categories representing two significant processes—immune system regulation and apoptosis (Table 3). GO enrichment of the highly expressed Schizophrenia genes yielded a much different set of processes, mostly implicating cellular morphogenesis, but none involving the immune response (Table 4). The epilepsy dataset did not enrich for any significant functions when considering those genes that were highly expressed. This suggests that ASD-implicated genes with no or low expression in the developing brain may obscure functional pathway analysis, which otherwise implicates cytokine signaling.

**Table 3:** GO enrichment analysis of highly expressed Autism genes.

GO Term	Description	P-value
GO:0002682	regulation of immune system process	0.0001
GO:0006915	apoptosis	0.0009
GO:0012501	programmed cell death	0.0009
GO:0031347	regulation of defense response	0.0009

**Table 4:** GO enrichment analysis of highly expressed Schizophrenia genes.

GO Term	Description	P-value
GO:0048812	neuron projection morphogenesis	0.00001
GO:0032990	cell part morphogenesis	0.00002
GO:0048858	cell projection morphogenesis	0.00002
GO:0032989	cellular component morphogenesis	0.00007
GO:0007409	axonogenesis	0.00007
GO:0090066	regulation of anatomical structure size	0.00008
GO:0051129	negative regulation of cellular component organization	0.00063
GO:0032535	regulation of cellular component size	0.00065
GO:0010721	negative regulation of cell development	0.00083
GO:0007417	central nervous system development	0.00083
GO:0030030	cell projection organization	0.00084
GO:0031344	regulation of cell projection organization	0.00096

### Network Analysis

Next, we set out to determine if the genes we identified as being highly expressed in the developing brain implicate different functional networks as compared to all genes associated with these diseases. We utilized integrated gene-network analysis using the curated Ingenuity Pathway Analysis (IPA) database. Initially we searched for canonical pathways for each disorder, comparing the highly expressed gene sets to all disease-associated genes (Tables 5, 6, 7). This analysis implicated many new canonical pathways from the set of highly expressed genes not seen in the full dataset analysis. For Autism, this included corticotrophin releasing hormone signaling, g-protein and phospholipase C signaling, and neutrophil cytokine signaling. The new pathways implicated in Schizophrenia included synaptic long-term potentiation and axon guidance signaling, and in Epilepsy semaphorin signaling and the splicesome cycle. Interestingly, there are no canonical pathways shared between the three disorders when the entire set of implicated genes is considered, but analysis of the *highly expressed sets* implicates Reelin Signaling in Neurons as common to all three disorders. Further investigation of this pathway (Fig. S6) shows almost all molecules are implicated in at least one of these three neurodevelopmental disorders.

**Table 5:** Canonical Pathways implicated in ASD when considering all genes versus highly expressed genes.

Canonical Pathways Derived from All AutDB Genes	P-Value	Canonical Pathways Derived from 32 Highly Expressed ASD Genes	P-Value
Serotonin Receptor Signaling	4.4E-08	Corticotropin Releasing Hormone Signaling	4.5E-05
Reelin Signaling in Neurons**	5.4E-06	G-Protein Coupled Receptor Signaling**	2.5E-04
HER-2 Signaling in Breast Cancer	5.0E-05	Role of NFAT in Cardiac Hypertrophy	3.1E-04
cAMP-mediated Signaling**	6.9E-05	Reelin Signaling in Neurons**#	3.7E-04
G-Protein Coupled Receptor Signaling**	7.6E-05	Factors Promoting Cardiogenesis in Vertebrates	5.0E-04
Virus Entry via Endocytic Pathways**	1.7E-04	α-Adrenergic Signaling	5.1E-04
Macropinocytosis Signaling	1.8E-04	cAMP-mediated Signaling**	5.1E-04
Axonal Guidance Signaling	2.2E-04	Virus Entry via Endocytic Pathways**	5.5E-04
PTEN Signaling	3.8E-04	G Beta Gamma Signaling	5.9E-04
GABA Receptor Signaling	4.3E-04	Phospholipase C Signaling	7.2E-04
Glioblastoma Multiforme Signaling	5.9E-04	Cholecystokinin/Gastrin-mediated Signaling	7.8E-04
PI3K/AKT Signaling	9.1E-04	fMLP Signaling in Neutrophils	9.5E-04

**indicates the pathway was implicated in both sets. #indicates the pathway was common to all three disorders.

**Table 6:** Canonical Pathways implicated in Schizophrenia when considering all genes versus highly expressed genes.

Canonical Pathways Derived from All Schizophrenia Genes	P-Value	Canonical Pathways Derived from 212 Highly Expressed Schizophrenia Genes	P-Value
Glutamate Receptor Signaling**	1.0E-32	Glutamate Receptor Signaling**	1.3E-12
Amyotrophic Lateral Sclerosis Signaling**	4.0E-23	Reelin Signaling in Neurons**#	4.4E-09
Neuropathic Pain Signaling In Dorsal Horn Neurons	7.9E-22	cAMP-mediated Signaling	1.5E-08
CREB Signaling in Neurons**	1.0E-21	14-3-3-mediated Signaling	4.3E-08
Role of Macrophages, Fibroblasts and Endothelial Cells in Rheumatoid Arthritis	1.3E-20	Axonal Guidance Signaling	6.6E-08
Role of Osteoblasts, Osteoclasts and Chondrocytes in Rheumatoid Arthritis	1.3E-19	p70S6K Signaling	8.7E-08
G-Protein Coupled Receptor Signaling	4.0E-18	CREB Signaling in Neurons**	9.1E-08
Human Embryonic Stem Cell Pluripotency	1.3E-17	Synaptic Long Term Potentiation	1.9E-07
Serotonin Receptor Signaling	5.0E-17	Myc Mediated Apoptosis Signaling	1.1E-06
Glucocorticoid Receptor Signaling	7.9E-17	Amyotrophic Lateral Sclerosis Signaling**	1.1E-06

**indicates the pathway was implicated in both sets. #indicates the pathway was common to all three disorders.

**Table 7:** Canonical Pathways implicated in Epilepsy when considering all genes versus highly expressed genes.

Canonical Pathways Derived from All Epilepsy Genes	P-Value	Canonical Pathways Derived from 42 Highly Expressed Epilepsy Genes	P-Value
GABA Receptor Signaling**	4.7E-09	Reelin Signaling in Neurons**#	1.3E-06
Neuropathic Pain Signaling In Dorsal Horn Neurons	2.1E-06	GABA Receptor Signaling**	2.8E-04
Reelin Signaling in Neurons**	2.6E-05	Semaphorin Signaling in Neurons	7.1E-03
β-alanine Metabolism	2.2E-04	Spliceosomal Cycle	7.2E-03
Glutamate Receptor Signaling**	7.4E-04	Glutamate Receptor Signaling**	1.1E-02
Calcium Signaling	8.5E-04		
Cellular Effects of Sildenafil (Viagra)	1.1E-03		
Butanoate Metabolism	2.7E-03		
Hepatic Cholestasis	5.5E-03		
Glutamate Metabolism	6.0E-03		

**indicates the pathway was implicated in both sets. #indicates the pathway was common to all three disorders.

**Figure 3:**
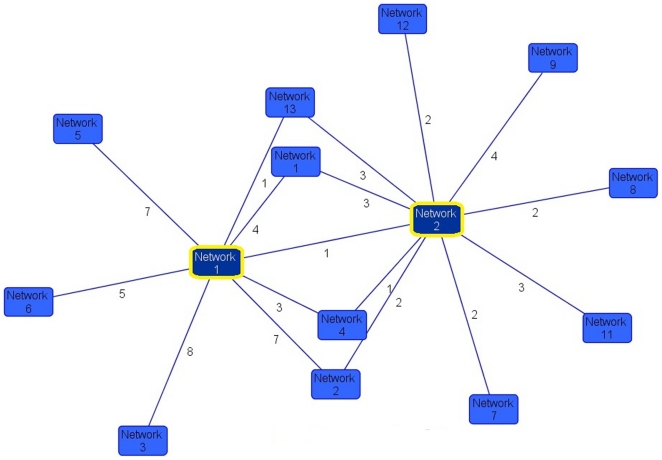
Overlapping gene-networks in ASD. Networks 1 and 2 (yellow border) were derived from the highly expressed ASD gene set. All other networks were derived from the set of all ASD-implicated genes. Orphaned networks (no edges) were excluded. Edge values represent number of interactions between networks.

**Figure 4:**
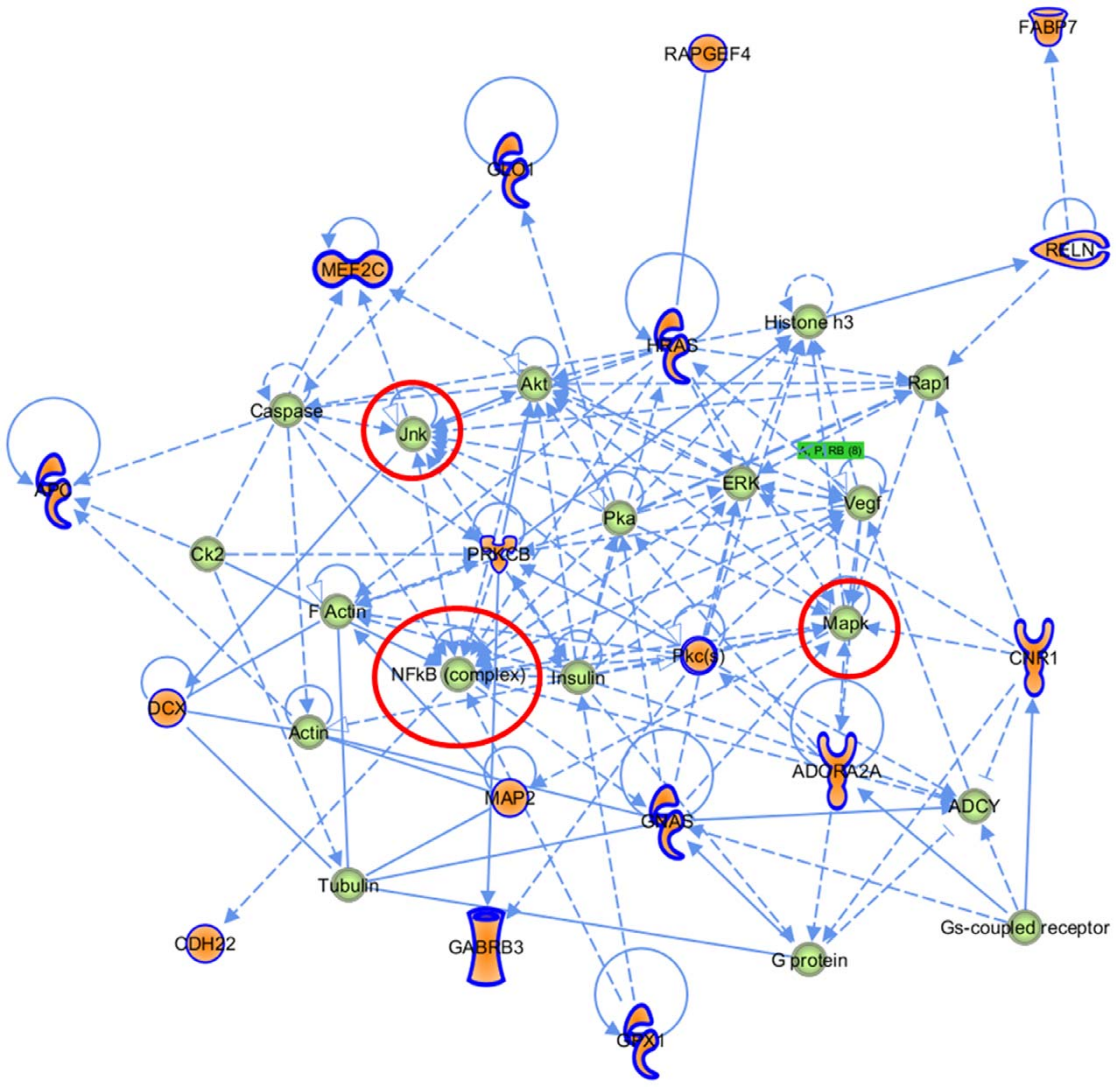
Network 1 derived from the ASD highly expressed gene set. Orange genes are those present in AutDB that are highly expressed.

**Figure 5:**
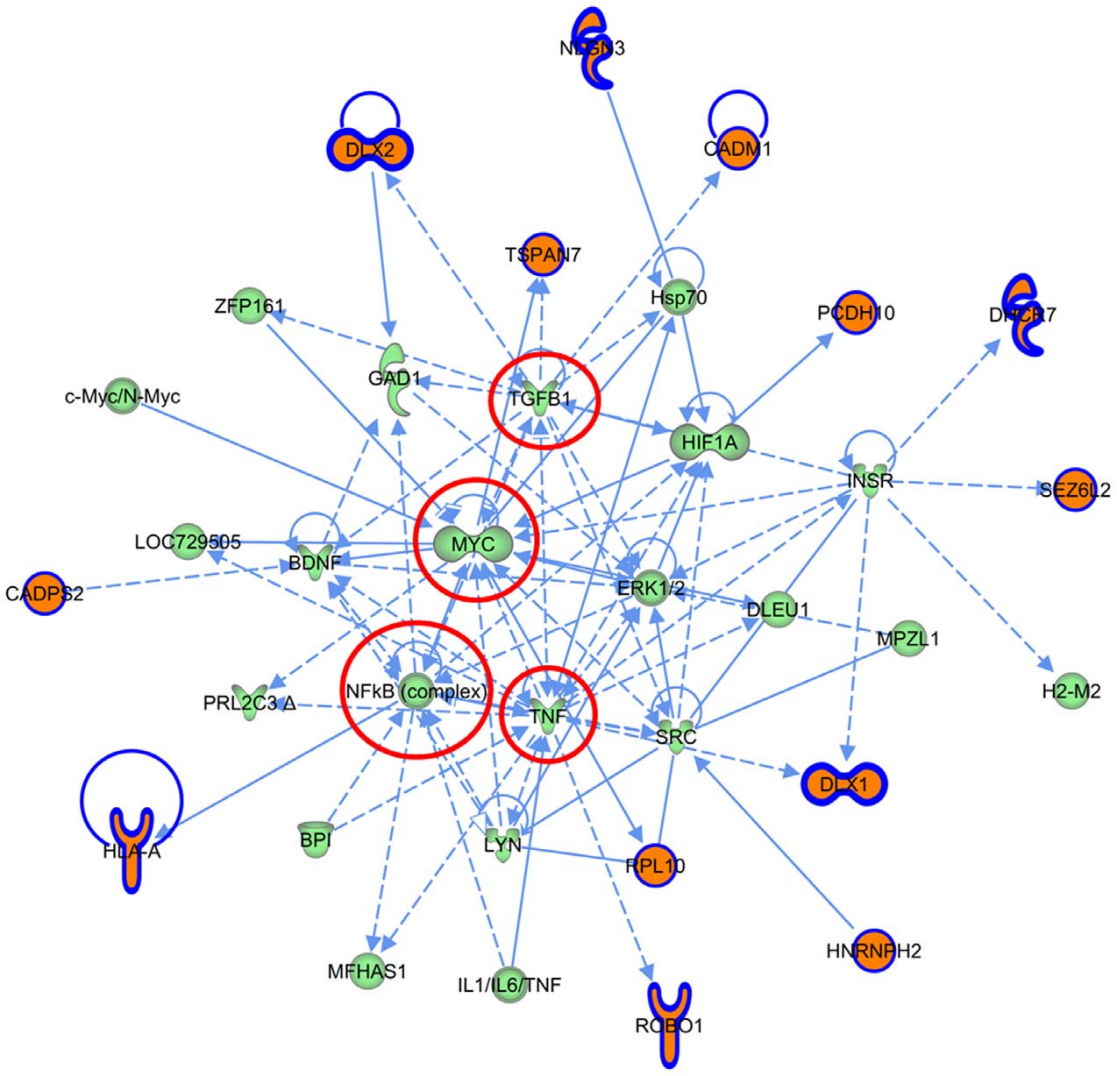
Network 2 derived from the ASD highly expressed gene set. Orange genes are those present in AutDB that are highly expressed.

Unbiased gene-network analysis was then constructed in IPA, to identify connectivity networks derived from the enriched gene set compared to those derived from all Autism-associated genes. Overlaying derived networks based on connectivity revealed that the two networks constructed from the highly expressed ASD genes are central to all networks obtained from all ASD-associated genes (Figure 3). In the first central network (Figure 4), *NFκB*, *Jnk*, and *Mapk* are hubs. Network 2 from the highly enriched set also contains *NFκB* as a hub, in addition to *Tnf*, *TgfB1* and *Myc* (Figure 5). Taken together, these enriched networks, which are the most inter-connected of all ASD-derived networks, have at their core fundamental cytokine signaling molecules not previously implicated as ASD susceptibility loci. These may serve as potential final common pathways through which the heterogeneous ASD-implicated genes ultimately converge. Moreover, this represents a third, independent level of analysis whereby the highly expressed ASD genes implicate immune signaling pathways that are not apparent when the full set of ASD-associated genes is considered.

Similar analysis comparing ASD-associated gene networks specific to brain regions did not result in a significant clustering by region, nor were there central network nodes (Fig. S7). Considering only genes expressed highly during gestational time points, we did not observe any new pathways or networks not already implicated using all time points. Gene-network analysis of the Epilepsy and Schizophrenia gene sets did not result in centrality of the highly expressed networks as we observed in ASD (Figures S8 and S9), perhaps reflecting the less heterogeneous nature of these disorders.

### Correlating Gene Transcription with Cell-type Specific Protein Expression

Next, we were interested in correlating our ASD gene transcriptome results with protein expression levels in a cell-type specific manner. To do so, we mined the Human Protein Atlas database for the 32 highly expressed Autism genes (see Methods, Table 8). We were surprised to find that *many highly expressed ASD genes are mainly detected in glia not neurons*, and/or in *specific layers of the cerebellum*. A similar proportion of genes exhibit neuron-specific protein expression. We also observed that *Gnas*, a complex locus known to be imprinted and express antisense and non-coding transcripts [44], does not appear to make detectable protein in the CNS, yet is one of the 9 most highly expressed ASD transcripts in the NIMH atlas. *Fabp7*, which we noted was *the* most highly expressed ASD-associated gene, is only detected in glia. Moreover, *Cnr1*—the one highly expressed gene shared by all three disorders—is most highly expressed in glial cells and the molecular layer of the cerebellum. These results suggest future investigation of cell-type specific expression in ASD will be an important undertaking, and consideration of non-coding RNAs in ASD pathogenesis is warranted as well.

**Table 8:** Cell-type specific protein expression of highly expressed ASD genes from the Human Protein Atlas database.

	Neurons			Glia			Cerebellum		
	Cortex	Hipp	Lat Vent	Cortex	Hipp	Lat Vent	Purkinje	Granular	Molecular
FABP7	−	−	−	++	+++	++	−	−	++
GNAS	−	−	−	−	−	−	−	−	−
GPX1	+	+	−	++	++	++	++	−	−
HNRNPH2	+++	+++	+++	+++	++	++	++	+++	+++
HRAS	+++	+++	+++	+++	+++	+++	−	+++	+++
PDZD4	++	+	++	+	+	+++	++	−	++
RPL10	+++	+++	+++	+++	++	++	+++	++	+++
TSPAN7	+++	++	++	−	−	−	−	−	−
MAP2	+++	+++	+++	−	−	−	+++	+++	+++
PRKCB	++	++	++	−	−	+	++	+++	++
MEF2C	+++	+++	+++	+++	+++	+++	+++	+	+++
RAPGEF4	+++	++	−	++	+	++	+++	+	++
APC	+	+	++	++	++	++	++	++	+++
DCX	+++	+	+	++	++	++	++	++	+++
RIMS3	+	+	+	−	−	−	+	++	−
ROBO1	++	++	++	+++	++	++	++	++	++
GLO1	++	++	++	++	++	++	−	−	+
DLX2	+++	++	++	+	+	+	++	+	−
CNR1	++	+	+	+++	+++	+++	+	++	+++
PCDH10	++	++	+	+++	+++	+++	+	++	+++
NLGN3	++	+++	+	−	−	−	+++	+++	−
RELN	+	+	+	−	−	−	+	−	−
CADM1	−	−	−	−	−	−	−	+++	−
CDH22	+	−	−	+	+	+	−	++	−

Data is reported as presented in the Atlas: +++ for “strong” expression, ++ for “moderate,” + for “weak,” and − for “negative.” These highly expressed ASD genes were not present in the database: *Sez6l2, Gabrb3, Hsd11b1l, Hla-A, Dlx1, Adora2a, Cadps2*, and *DHCR7*. Lat vent = Lateral Ventricle, Hipp = Hippocampus.

### Analysis of ASD Transcriptome Data

Lastly, we were interested in considering our findings in the context of the major three published transcriptomics studies on ASD brain tissue [30], [31], [32]. Two of these have implicated immune alterations in ASD brain as compared to controls. We examined the results of all three studies to determine how many of the dysregulated genes reported were ASD implicated genes present in AutDB. Surprisingly, in each study only ∼5% of genes that are significantly different between ASD and control brains were previously implicated in ASD (Table 9). This underscores the importance of our findings on ASD-implicated genes, as both our approach and whole-transcriptomics studies implicate immune signaling pathways, even though most ASD-implicated genes we profiled are not dysregulated in ASD brain tissue.

**Table 9:** Correlation of AutDB genes with published transcriptome studies in ASD brain.

	Garbet *et al* 2008	Voineagu *et al* 2011	Purcell *et al* 2001
Brain tissue studied	STC	STC, PFC, Cerebellum	Mainly Cerebellum
# of samples	6 ASD, 6 Ctrls	29 ASD, 29 Ctrls (cortex) 11ASD, 10 Ctrl (cerebellum)	10 ASD, 23 Ctrls
Transcriptome Profiling Method	U133 Plus 2.0 GeneChip (Affymetrix)	Ref8 v3 Array (Illumina)	Clontech Array and UniGEM V2 Array
# of genes dysregulated in ASD	130	444	30
Main findings	↑ Immune-related genes	Genes converge on immune and synapse modules	↑ AMPA-type glutamate receptors
	↓ Genes involved in neuronal development		
Dysregulated genes in AutDB	4/130 (3%)^1^	21/444 (4.7%)^2^	1/31 (3.2%)^3^

1 SDC2, SLC9A9, DLX1, AHI1.

2 CD44, CDH10, DLX1, DPP6, GABRB3, HLA-A, KCNMA1, MET, NOS2A, PRKCB1, PTGS2, SCN1A, SLC25A12, NLGN4Y, CADM1, A2BP1, AHI1, PCDH10, PDZD4, CADPS2, SLC9A9.

3 CNR1.

STC = Superior temporal cortex, PFC = Pre-frontal cortex, cere = cerebellum.

## Discussion

In an attempt to integrate the genomic heterogeneity underlying the complex etiologies of common neurodevelopmental disorders, we report here the analysis of expression from all implicated genes in Autism, Schizophrenia, and Epilepsy using next-generation transcriptome sequencing in the developing human brain. Sakai *et al* recently constructed a protein interactome network using a yeast two-hybrid screen on a subset of ASD candidate genes [45], but to our knowledge, no study has yet attempted to derive molecular pathways underlying ASD by investigating as large of a set of ASD candidate genes.

To do so, we first described gene ontology, canonical pathways, and interactome networks for all genes implicated in ASD that are cataloged in the database AutDB. Then, based on the argument that differential expression of ASD-implicated genes may underlie the clinical and genetic heterogeneity, we developed a biologically relevant methodology to extract a subset of highly expressed ASD-implicated genes from the NIMH Transcriptional Atlas of Human Brain Development. We found that interactome analysis placed the two networks derived from highly expressed ASD candidate genes at the center of all ASD gene networks. Closer inspection of these networks revealed *NFκB*, *Jnk*, *MapK*, *TNF*, *TGF-B*, and *Myc* as central hubs. These central networks were supported by evidence at two other levels of our analysis (Gene ontology and canonical pathways). Taken together, our findings integrate a large set of genes implicated in ASD and suggest that they may converge onto classical cytokine signaling pathways. While other transcriptomics studies on ASD tissue have implicated immune system signaling in ASD pathogenesis, our findings suggest that the ASD-implicated genes *themselves* may also be related to these functions.

Interestingly, there is also mounting evidence at the cellular and tissue levels that more in depth investigation of an immune component is warranted in ASD [46]. For instance, multiple studies have demonstrated altered cytokine profiles in ASD patients [47], [48], and altered *TGF-B* concentration in serum and CSF correlates with disease severity [49]. Others have described various autoimmune phenomena including autoantibodies to neural antigens and maternal-fetal cross-reactive neural antibodies [50]. There is also indication of altered innate cellular immunity in ASD, such as differences in gene expression and altered response to immunostimlulatory ligands in both natural killer and monocytic cells from ASD patients [51], [52]. Post-mortem brain tissue from ASD patients shows increased microglial density in grey matter, an activated morphology, and secretion of a cytokine profile consistent with a pro-inflammatory state, most prominent in the cerebellum [53], [54]. Moreover, microglia from MeCP2- null mice—a model of the Autism Spectrum Disorder Rett Syndrome—produce a conditioned media that damages synaptic connectivity via a glutamate-excitotoxicity mechanism [55]. While all of this work provides post-hoc evidence for altered immune response in ASD, our results suggest a direct link between implicated genes in ASD and molecular pathways involved in immune signaling.

This considerable attention to the immune response in previous ASD research has resulted in two prevailing theories: one suggests exogenous factor(s) stimulate neuro-inflammation during development, while the other postulates autoimmune activation causes ASD pathology [56], [57]. However, it is equally possible—as our results support—that the mutations described in ASD result in aberrant signaling regulation of immune cells during neurodevelopment. This could result in cell-autonomous activation and/or improper response to otherwise nominal stimuli, such as occurs in the autoinflammatory syndromes [58]. Alternatively, as glia are increasingly implicated in normal formation of synaptic connectivity [24]—and we have demonstrated a significant proportion of ASD-implicated genes appear to be glial-specific—it is possible that genomic aberrations ultimately funnel through core signaling pathways of glial cells to disrupt formation of neural networks independent of an inflammatory mechanism. In support of this notion, a number of recent reports have demonstrated that these same cytokine signaling pathways are central to proper brain development [59], [60]. Furthermore, signaling through the NFkB pathway has been shown to be important in synaptic plasticity independent of an inflammatory mechanism [61].

Moreover, two of three genome-wide expression studies in Autism brain tissue conclude that the most prominent transcriptome changes are related to neuro-immune disturbances. In the Garbett *et al* study, the most significant functional pathway implicated was NFκB signaling [31]. The most comprehensive transcriptomics study of ASD post-mortem brain to date (Voineagu *et al*) concludes that one of two significant co-expression networks is involved in immune function [32]. While our results are only a first step in linking common molecular interaction pathways to the underlying genetic heterogeneity of ASD, they provide integrated genomic evidence, which is supported by these transcriptomics, cell, and tissue level studies that further investigation into cytokine signaling in ASD is needed.

In summary, we report the spatial and temporal expression profile of genes implicated in Autism Spectrum Disorders, in addition to the genetically and phenotypically related neurodevelopmental disorders Schizophrenia and Epilepsy. We found a large proportion of implicated genes are not expressed in the developing human brain, and a significant number appear to be mainly expressed in glial cells. Integrated gene-network analysis, gene ontology enrichment, and canonical pathways investigation of a subset of highly expressed ASD genes all implicate central immune signaling pathways as common to the heterogeneous interactome of the implicated genes. This work serves as a framework to link the genetic findings in ASD with transcriptome, cell, and tissue level evidence for altered immune functions in ASD patients.

## Methods

### Neurodevelopmental Disorder Databases

AutDB [34] and CarpeDB [62] are updated, publically downloadable databases that catalog genes implicated/associated with Autism and Epilepsy, respectively (Table S1). SZGene [63] is a similar searchable database, and we obtained datafiles directly from the curators (Table S1). In all three databases, some implicated regions are provisional loci, non-coding RNAs, pseudogenes, or otherwise not included in the NIMH Transcriptional Atlas of Human Brain Development and, therefore, were not considered. All genes used in this study and how they were implicated (e.g. GWAS, functional, etc), with corresponding references, are documented in Table S1.

### NIMH Transcriptional Atlas of Human Brain Development

The NIMH Transcriptional Atlas of Human Brain Development (www.developinghumanbrain.org) was accessed on 2/16/2011, and the raw Gene Matrix. csv datafile was downloaded. We re-organized the data so that rows are genes and columns are developmental time points subdivided according to brain region. While the Atlas contains data for 16 different brain regions, we narrowed our focus to those 11 that were most relevant to autism [20], [41]: Dorsolateral Prefrontal Cortex (DLPC), Ventrolateral Prefrontal Cortex (VLPC), Medial Prefrontal Cortex (MPC), Orbital Prefrontal Cortex (OPC), Posterior Superior Temporal Cortex (PSTC), Inferior Lateral Temporal Cortex (ILTC), Hippocampus (Hipp), Amygdala (Amyg), Striatum (Stri), Cerebellum (Cere), and Primary Motor Cortex (PMC). Genes in AutDB, CarpeDB, and SZGene were parsed from the full database to create disease-specific expression databases (Tables S2, S3, S4). Our analysis only considered time points up to 23 years old.

### Gene Expression Analysis

Expression values were divided into quintiles and given corresponding colors for heat map creation (<20 RPKM, 20–40 RPKM, 40–60 RPKM, 60–80 RPKM, >100 RPKM). Genes were then assigned to one of five tiers within each brain region based on their highest level of expression across all time points, in a conservative attempt to analyze the expression data qualitatively. For example, if a gene is expressed at 150 RPKM at 24 weeks gestation (wg) and at 80 RPKM for all other time points, it is placed in the >100 RPKM tier. Based on results from established housekeeping genes (see Results), we considered a gene to be differentially expressed if it crossed more than three tiers. Because of this, genes in the top three tiers were considered to be “highly expressed,” and were the focus of our subsequent analysis (Tables S2, S3, S4, “Highly Expressed Genes” tab).

### Gene Ontology Enrichment Analysis

To test if a subset of genes implicated different Gene Ontology categories than a background set of all genes, we employed the Gene Ontology Enrichment Analysis and Visualization tool [64], accessed at http://cbl-gorilla.cs.technion.ac.il/. We specified the organism as *Homo sapiens*, chose the option for two unranked lists of genes, and set the p-value threshold to 0.01.

### Ingenuity Pathway Analysis

Integrated gene-network analysis for the AutDB, CarpeDB, and SZGene sets and on the highly expressed subsets were generated by Ingenuity Pathways Analysis (Version 8.8, Ingenuity® Systems, www.ingenuity.com). Each gene identifier was mapped to its corresponding gene object in the Ingenuity Pathways Knowledge Base. The gene lists were overlaid onto a global molecular network developed from information contained in the Ingenuity Pathways Knowledge Base. These focus gene networks were then algorithmically generated based on their connectivity.

Canonical pathways analysis identified the pathways from the Ingenuity Pathways Analysis library of canonical pathways that were most significant to the data set. The significance of the association between the data set and the canonical pathway was measured in two ways: i) A ratio of the number of molecules from the data set that map to the pathway divided by the total number of molecules that map to the canonical pathway and ii) Fisher's exact test was used to calculate a p-value determining the probability that the association between the genes in the dataset and the canonical pathway is explained by chance alone. A p-value of less than 0.01 was considered significant. For comparison analysis between all disease genes and highly expressed genes, Benjamini-Hochberg multiple testing correction was used to calculate p-values, with 0.01 set as a significance threshold.

Functional Network Analysis identified the biological interactions that were most significant to the molecules in the network. The network molecules associated with biological functions and/or diseases in Ingenuity's Knowledge Base were considered for the analysis. Right-tailed Fisher's exact test was used to calculate a p-value determining the probability that each biological function assigned to that network is due to chance alone, with a threshold of 0.01 set for significance. A graphical representation of the molecular relationships between molecules was generated. Molecules are represented as nodes, and the biological relationship between two nodes is represented as an edge (line). All edges are supported by at least one reference from the literature. Nodes are displayed using various shapes that represent the functional class of the gene product. Edges are displayed as either solid or broken lines to describe the nature of the relationship between the nodes (solid for direct interaction, broken for an indirect interaction).

### Human Protein Atlas

To compare expression data at the transcriptome level to protein-level expression, we accessed the Human Protein Atlas [35] at http://www.proteinatlas.org/. The Human Protein Atlas is a publicly available database cataloging the distribution of proteins in different normal human tissues, cancer types, and cell lines via validated antibody analysis. The data includes immunohistochemisty, Western blot analysis and, for a large fraction, a protein array assay and immunofluorescent based confocal microscopy. We utilized the reported levels of antibody staining as given, except for genes that contained annotated expression results, which we reported instead.

## Supporting Information

Figure S1 **Expression profile of the 15 most constantly expressed genes identified by Hsiao** ***et al*** **.**
(XLS)

Figure S2 **Expression profile of 10 cannonical housekeeping genes.**
(XLS)

Figure S3 **Expression profiles of 5 tissue-specific Intermediate Filament Genes.** NEFL is specific to neurons (Neurofilament, Entrez ID 4747), GFAP is specific to Glia (Glial Fibriallary Acidic Protein, Entrez ID 2670), VIM is specific to mesoderm derivatives (Vimentin, Entrez ID 7431), DES is specific to muscle (Desmin, Entrez ID 1674), and KRT1 (Keratin, Entrez ID 3848) is specific to epithelium.
(XLS)

Figure S4 **Expression profile of Cannabinoid Receptor 1 and 2.**
(XLS)

Figure S5 **Expression profile of the most highly expressed ASD-implicated genes.**
(XLS)

Figure S6 **Schematic diagram of the canonical pathway “Reelin Signaling in Neurons.”** Genes implicated in ASD, Epilepsy, and Schizophrenia are indicated with shading. Grey shading indicates the gene is implicated in more than one of these disorders, yellow is specific to Schizophrenia and Pink specific to ASD.
(TIF)

Figure S7 **Overlay of ASD interactome networks by brain region for the 32 highly expressed genes.** There was no significant clustering of networks by brain region. Dark blue = Amygdala, Light Blue = Motor, Turquoise = Striatum, Black = Combined frontal, Grey = Combined temporal, Beige = Hippocampus, Dark Red = Cerebellum.
(TIF)

Figure S8 **Overlay of Schizophrenia interactome networks.** Dark blue networks are from the highly expressed enriched set, light blue networks from all Schizophrenia genes analyzed. No central networks were apparent.
(TIF)

Figure S9 **Overlay of Epilepsy interactome networks.** Dark blue networks are from the highly expressed enriched set, light blue networks from all Epilepsy genes analyzed. No central networks were apparent.
(TIF)

Table S1 **Genes from AutDB, SZGene, and CarpeDB.**
(XLSX)

Table S2 **Expression heat map of AutDB genes.**
(XLSX)

Table S3 **Expression heat map of SZGene genes.**
(XLSX)

Table S4 **Expression heat map of CarpeDB genes.**
(XLSX)

Table S5 **Temporal Expression Profiles by Region for all 32 Highly Expressed ASD genes.**
(XLSX)
