# Supplementary figures and images for: Expression Profiling of Autism Candidate Genes during Human Brain Development Implicates Central Immune Signaling Pathways
Source: PLoS One. 2011 Sep 15;6(9):e24691. doi: 10.1371/journal.pone.0024691 (PMC3174192; doi:10.1371/journal.pone.0024691)

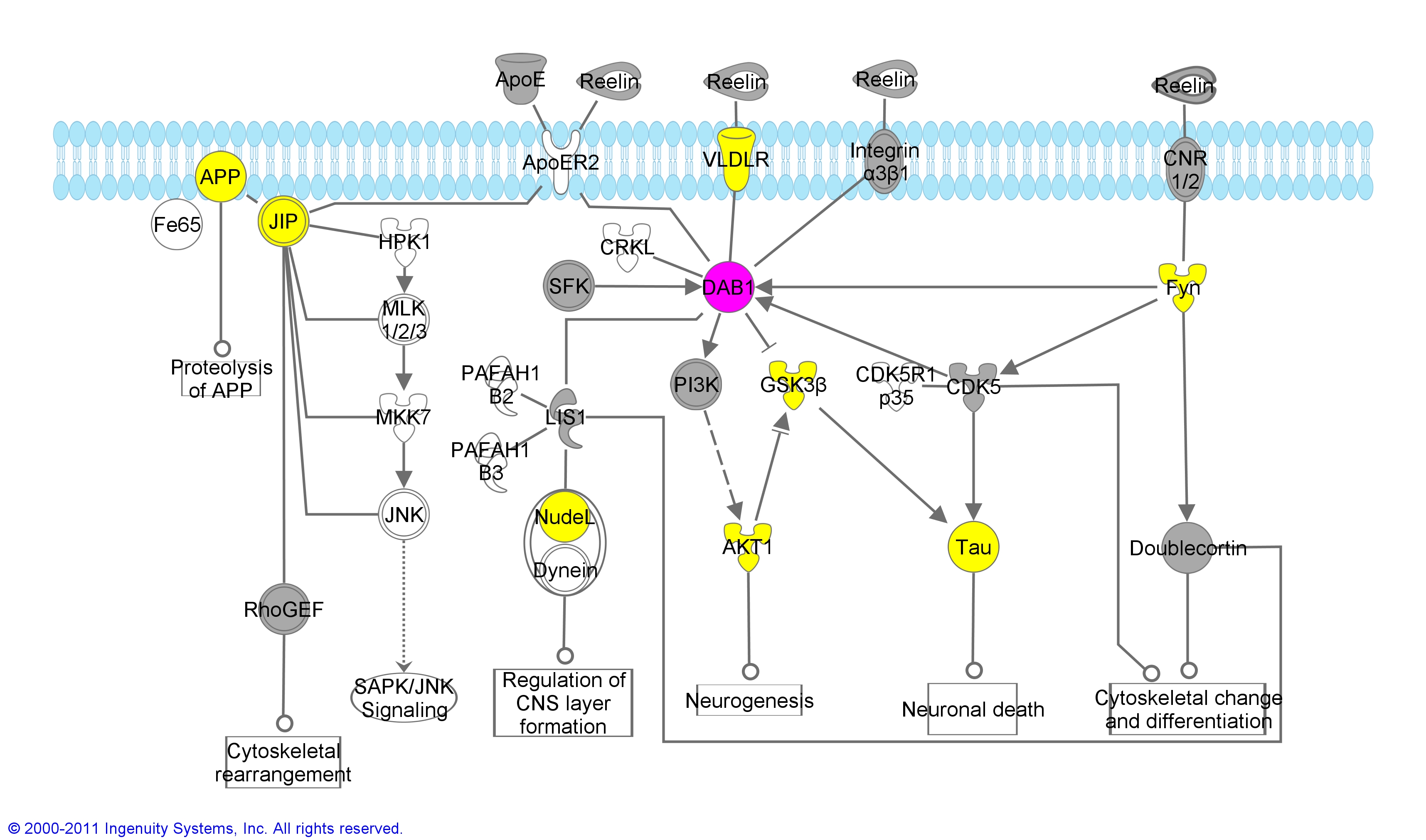

Supplement: Figure S6 — Schematic diagram of the canonical pathway “Reelin Signaling in Neurons.” Genes implicated in ASD, Epilepsy, and Schizophrenia are indicated with shading. Grey shading indicates the gene is implicated in more than one of these disorders, yellow is specific to Schizophrenia and Pink specific to ASD. (TIF) [file pone.0024691.s006.tif]

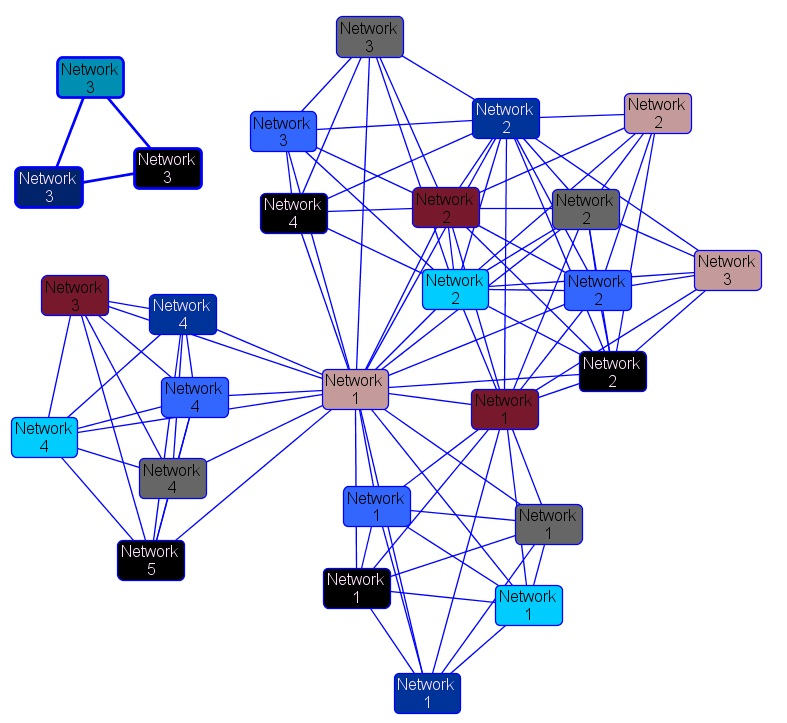

Supplement: Figure S7 — Overlay of ASD interactome networks by brain region for the 32 highly expressed genes. There was no significant clustering of networks by brain region. Dark blue = Amygdala, Light Blue = Motor, Turquoise = Striatum, Black = Combined frontal, Grey = Combined temporal, Beige = Hippocampus, Dark Red = Cerebellum. (TIF) [file pone.0024691.s007.tif]

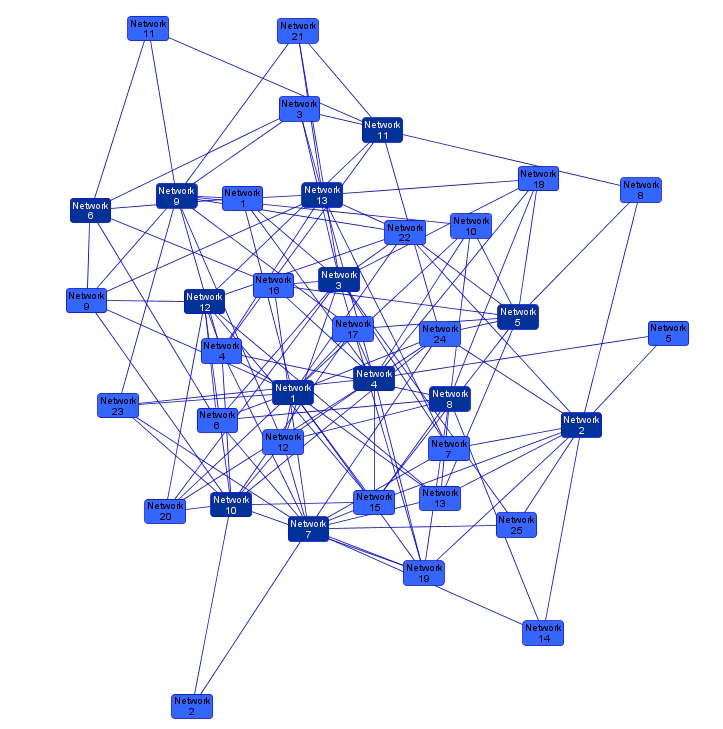

Supplement: Figure S8 — Overlay of Schizophrenia interactome networks. Dark blue networks are from the highly expressed enriched set, light blue networks from all Schizophrenia genes analyzed. No central networks were apparent. (TIF) [file pone.0024691.s008.tif]

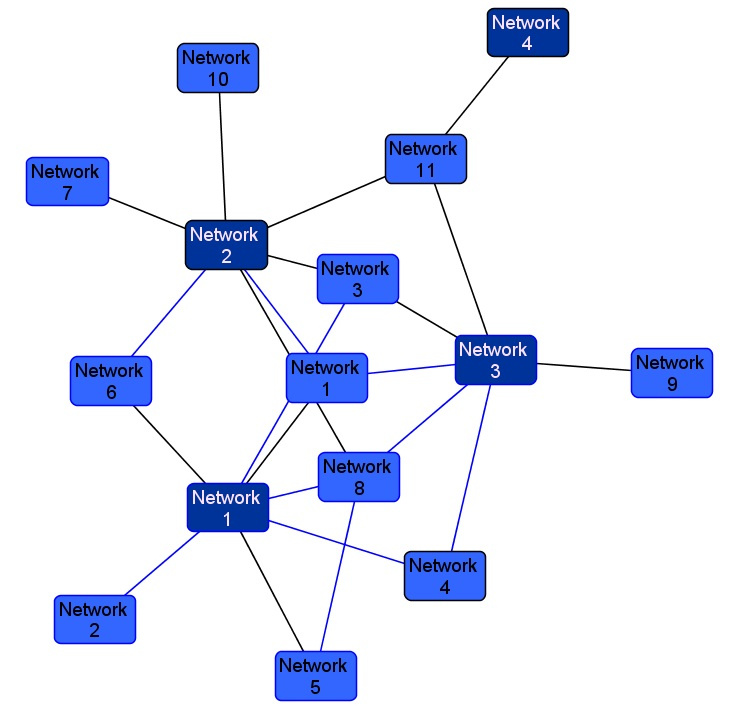

Supplement: Figure S9 — Overlay of Epilepsy interactome networks. Dark blue networks are from the highly expressed enriched set, light blue networks from all Epilepsy genes analyzed. No central networks were apparent. (TIF) [file pone.0024691.s009.tif]
